# Cytogenetic analyses using C-banding and DAPI/CMA3 staining of four populations of the maize weevil *Sitophilus zeamais* Motschulsky, 1855 (Coleoptera, Curculionidae)

**DOI:** 10.3897/CompCytogen.v9i1.4611

**Published:** 2015-03-31

**Authors:** Alexandra A. da Silva, Lucas S. Braga, Raul Narciso C. Guedes, Mara G. Tavares

**Affiliations:** 1 Departamento de Biologia Geral, Universidade Federal de Viçosa, Viçosa, Minas Gerais, 36570-000, Brazil; 2 Departamento de Entomologia, Universidade Federal de Viçosa, Viçosa, Minas Gerais, 36570-000, Brazil

**Keywords:** Heterochromatin, B chromosomes, C-banding, karyotype, fluorochromes

## Abstract

Cytogenetic data avalaible for the maize weevil *Sitophilus zeamais* Motschulsky, 1855 (Coleoptera: Curculionidae), one of the most destructive pests of stored cereal grains, are controversial. Earlier studies focused on single populations and emphasized chromosome number and sex determination system. In this paper, the karyotypes of four populations of *Sitophilus zeamais* were characterized by conventional staining, C-banding and sequential staining with the fluorochromes chromomycin-A_3_/4-6-diamidino-2-phenylindole (CMA_3_/DAPI). The analyses of metaphases obtained from the cerebral ganglia of last instar larvae and the testes of adults showed that the species had 2n = 22 chromosomes, with 10 autosomal pairs and a sex chromosome pair (XX in females and Xy_p_ in males). Chromosome number, however, ranged from 2n = 22 to 26 due to the presence of 0–4 supernumerary chromosomes in individuals from the populations of Viçosa, Unai and Porto Alegre. With the exception of the Y chromosome, which was dot-like, all other chromosomes of this species were metacentric, including the supernumeraries. The heterochromatin was present in the centromeric regions of all autosomes and in the centromere of the X chromosome. The B chromosomes were partially or totally heterochromatic, and the Y chromosome was euchromatic. The heterochromatic regions were labeled with C-banding and DAPI, which showed that they were rich in AT base pairs.

## Introduction

Cytogenetic analyses are traditionally powerful tools for species characterization, identification and recognition of cryptic species, and the establishment of phylogenetic relationships and the evolutionary history of a species (Holecová et al. 2002, Rozek et al. 2004, Lachowska et al. 2004, 2006, 2008, 2009, Angus et al. 2011). Although such studies typically focus on single populations of different species, karyotype differences do exist among populations and may be potentially important for their divergence (e.g., Hsiao and Hsiao 1984).

Insect pest species present interesting cytogenetic challenges with potential practical consequences. Grain weevils (Coleoptera: Curculionidae) are a good example and include three important pest species of stored cereal grains (the granary weevil *Sitophilus granarius* (Linnaeus, 1875), the maize weevil *Sitophilus zeamais* Motschulsky, 1855, and the rice weevil *Sitophilus oryzae* (Linnaeus, 1763)), in addition to the tamarind weevil *Sitophilus linearis* (Herbst, 1797). These species belong to a 14 species genus of suspected Eurasian origin but with a current cosmopolitan distribution (Delobel and Grenier 1993). Grain weevils are also frequently found in archeological sites and provide important information on the human history of past urban environments, the origins of grain and likely dispersal routes, and routes of grain trade, in addition to the history of storage (Levinson and Levinson 1994, Kenway and Carrott 2006, Smith and Kenward 2011). Curiously, the evolutionary history of the grain weevils remains a matter of debate, with few cytogenetic studies and conflicting results.

The first cytogenetic analysis of a *Sitophilus* species described the karyotype of *Sitophilus granarius* with 12 pairs of chromosomes and the meioformula 5 + XX (Inkmann 1933, cited in Smith and Virkki 1978). Subsequently, a series of studies reviewed by Smith and Virkki (1978) found that the chromosome number for *Sitophilus oryzae* varied from 11 and 12 to 22, and the meioformulae were 5 + XX, 5 + X:XX, 10 + Xy or 10 + neoXY:XX. Furthermore, Barrion et al. (1988) reported that *Sitophilus oryzae* had 2n = 19 and a meioformula of n = 9 + XO, whereas both Zhi-Yua et al. (1989) and Moraes et al. (2003) described a karyotype with 22 chromosomes (2n = 20 + Xy). Takenouchi (1958, cited in Smith and Virkki 1978) also described the karyotype of *Sitophilus sasakii* (Takahashi, 1928) as containing 22 chromosomes (n = 10 + Xy).

For *Sitophilus zeamais*, the object of study of this work, Smith and Brower (1974) reported the presence of 22 chromosomes and a sex determination system of the neoXY type, plus the presence of 3–6 supernumerary chromosomes. Barrion et al. (1988), however, found 20 chromosomes (2n = 18 + XY), while Zhi-Yua et al. (1989) related the presence of 2n=22 chromosomes. More recently, Moraes et al. (2003) confirmed the occurrence of 22 chromosomes (2n = 20 + Xy) and the presence of 1 to 4 supernumerary chromosomes in this species.

However, these analyses were performed with meiotic chromosomes, obtained primarily through a squash of adult weevil testes because of the difficulty of obtaining mitotic metaphasic chromosomes (Petitpierre 1996), and focused on describing chromosome numbers and sex determination systems. Currently, however, more refined cytogenetic techniques, such as C-banding and base-specific fluorochrome staining, are used for cytogenetic characterization of different species of Curculionidae (Hsiao and Hsiao 1984, Holecová et al. 1997, 2002, 2013, Lachowska and Holecová 2000, Rozek et al. 2004, Lachowska et al. 2004, 2006, 2009). These techniques provide a better characterization of the karyotypes and reveal differences in the amount and location of heterochromatic regions between closely related species, as well as the AT and GC base pair constitution of these regions. Thus, these techniques may also be used to understand the karyotypic evolution of this group.

The current cytogenetic techniques indicated above show that most Curculionidae have a small amount of heterochromatin, located primarily in a centromeric/pericentromeric position (Holecová et al. 2002, Rozek et al. 2004, Lachowska et al. 2004, 2005). Some species, however, exhibited additional bands in the interstitial and/or in the telomeric regions, as was the case of *Acalles fallax* Boheman, 1844 and *Acalles echinatus* (Germar, 1824) (Lachowska et al. 2009). Additionally, with the use of fluorochromes, it was possible to show that the heterochromatin of most Curculionidae was AT rich (the C-bands coinciding with DAPI^+^ bands) (Lachowska 2008, Lachowska et al. 2008). Conversely, the CMA_3_^+^ bands were rarely found in the species of this family (Holecová et al. 2013).

Thus, because of the discrepancies with the karyotype of *Sitophilus zeamais* and the difficulties of working with meiotic chromosomes, the present work aimed to adapt methodologies to obtain mitotic chromosomes from cerebral ganglia cells of *Sitophilus zeamais* to characterize the karyotype of this species. We also analyzed four different populations of this species to verify the consistency of results and the existence of inter-population variations. It was also expected that this technique would be used in future studies as an easy, rapid and inexpensive method for the identification and separation of species of this genus. Additionally, we intended to develop a more detailed map of the location and composition of the heterochromatic regions in the genome of *Sitophilus zeamais*, with the use of C- and fluorochrome-banding techniques.

## Materials and methods

### Biological material

The larvae from four populations of *Sitophilus zeamais*, representing the occupation and migration route of this species in Brazil, were used. Because of the widespread distribution of this species in Brazil, populations from the north, south and center of the country were used. From the north of Brazil, a population from Cruzeiro do Sul (07°37'52"S and 72°40'12"W), a municipality located in the Acre State, was used. To represent the expansion of this species into more central regions, two populations with opposite locations in Minas Gerais State were used, one from Unai, in the northwest (16°21'27"S and 46°54'22"W), and the other from Viçosa, in the Zona da Mata Mineira (20°45'14"S and 42°52'55"W). From the south of Brazil, a population from Porto Alegre was selected (30°01'59"S and 51°13'48"W).

The populations were placed in glass containers (1.5 L) containing grains of maize and were stored in an environmentally controlled rearing room (25 ± 2 °C, 70 ± 10% relative humidity and a photoperiod of 12:12 h L:D). At the beginning of the analyses, the populations had been in culture for 6 months, 4 years, 1 and 6 months for the north, two central, and southern populations, respectively.

From preliminary tests, the last larval instar was determined to be the optimal stage for extraction of cerebral ganglia and preparation of slides because of the high number of cells in metaphase. As this stage developed inside the maize grain, the collection of larvae was preceded by inspection of the grain with X-ray equipment coupled to a 14-bit digital camera (Faxitron X-Ray Corp., Wheeling radiography equipment, IL, USA).

### Cytogenetic analyses

**a) Cerebral ganglia analyses**

The cerebral ganglia of individuals of the last larval stage were processed according to Imai et al. (1988) after incubation in a hypotonic solution of colchicine (1% sodium citrate plus 0.005% colchicine) for 1 h 45 min. After 24 h, the slides were stained with 4% Giemsa in Sörensen`s phosphate buffer pH 6.8, for 12 min. On average, 35 individuals of each population were analyzed.

The C-banding technique was performed according to Lachowska et al. (2005), with modifications at the time of the HCl treatment (0,3M, for 4 min) and the Ba(OH)_2_ incubation (3 min). The fluorochrome staining with DAPI/CMA_3_ was performed according to Schweizer (1980), with modifications related to the order of use of fluorochromes and the processing times (DAPI was used first for 30 min, followed by the CMA_3_ for 1 h). The use of distamycin was also omitted.

**b) Gonadal analyses**

To verify the behavior of the sex chromosomes and consequently confirm the sex determination system of the species, the analyses of the testes were performed according to Dias et al. (2012). Males were identified by morphology of the rostrum, which was smaller, thicker and more punctuated than the female rostrum (Khan and Musgrave 1968).

### Chromosomal Analyses

On average, 10 metaphases per slide were evaluated with an Olympus BX60 microscope coupled to an image capturing system (Image-Pro Plus^TM^, Version 6.3, Media Cybernetics®, 2009). The slides stained with fluorochromes (CMA_3_/DAPI) were analyzed with an epifluorescence light microscope using excitation filters WB (λ = 330–385 nm) and WU (λ = 450–480 nm) under oil immersion at 100× magnification. The chromosomes were classified according to Levan et al. (1964), and the karyotypes were mounted by pairing chromosomes in decreasing order of size.

## Results

**Figure 1.**
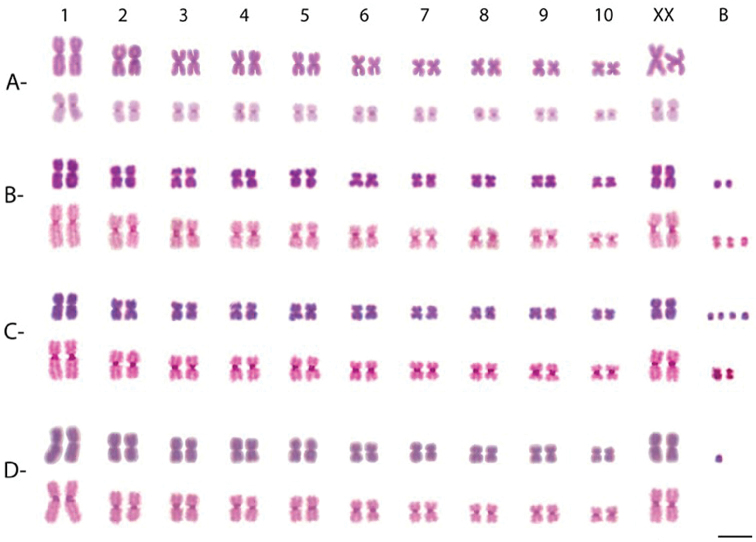
Karyotypes of females of *Sitophilus zeamais*. Populations from Cruzeiro do Sul (**A**), Unai (**B**), Porto Alegre (**C**), and Viçosa (**D**). Chromosomes show Giemsa staining and C-banding. B chromosomes are found in different populations. Bar = 10 μm.

**Figure 2.**
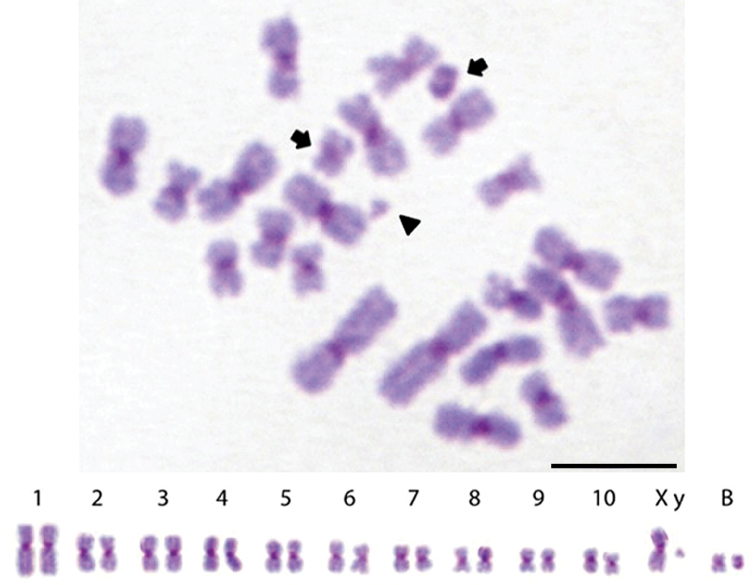
Metaphases and karyotypes of males of *Sitophilus zeamais* (2n = 22 + 2BS) from Porto Alegre. Chromosomes show C-banding. The arrows indicate the two types of B chromosomes, and the arrowhead indicates the Y chromosome. Bar = 10 μm.

The analyses of the cerebral ganglia showed that all populations of *Sitophilus zeamais* exhibited a karyotype with 20 autosomes and a pair of sex chromosomes, i.e., 2n = 22 chromosomes (Fig. 1). The autosomal chromosomes in the four populations exhibited metacentric morphology and a gradual reduction in size. The X chromosome of this species was also metacentric and relatively large, with an intermediate size compared to the first and second pair of autosomes (Fig. 1). The Y chromosome presented a dot-like morphology and was the smallest chromosome of the karyotype (Fig. 2). The sexual pair was identified with comparisons between male and female metaphases.

The analyses of the gonadal cells confirmed the chromosome number of this species, i.e., eleven chromosomal pairs. Additionally, the analyses showed the association of the “parachute” type between the sex chromosomes in metaphase I cells from male insects. Therefore, the meioformulae, n = 10 + XX and n = 10 + Xyp, were observed in females and males of *Sitophilus zeamais*, respectively.

All individuals in the population from Cruzeiro do Sul (AC) had 22 chromosomes, whereas the chromosome numbers ranged from 22 to 26 in the populations from Viçosa (MG), Unai (MG) and Porto Alegre (RS). These numerical changes occurred because of the presence of 0–4 B chromosomes (Table 1), which were found in cells of the same individual, in individuals of the same population and/or in individuals of different populations. These B chromosomes, in general, were larger than the Y chromosome and were easily distinguishable from the latter and from the autosomes and did not pair with each other or with the normal complement chromosomes.

**Table 1.** Frequency and types of B chromosomes found in the different populations of *Sitophilus zeamais*.

Population	Frequency of B Chromosomes (%)	Type of B Chromosome	N° slides analyzed
**Cruzeiro do Sul**	0%	-	35 females 2 males
**Unaí**	27,1% with 0 B 26,2% with 1B 24,8% with 2 Bs 21,9% with 3 Bs 0% with 4 Bs	Only type I	35 females
**Porto Alegre**	34,1% with 0 B 16,2% with 1 B 25,6% with 2 Bs 12,9% with 3 Bs 11,2% with 4 Bs	- Type I Type I Type I and II Type I Type I and II Type I	13 females 8 females 7 females 3 males 6 females 1 male 4 females
**Viçosa**	34,8% with 0 B 22,4% with 1 B 17,7% with 2 Bs 6,6% with 3 Bs 18,5% with 4 Bs	- Type I Type I Type I and II Type I Type I Type I and II	15 females 9 females 6 females 2 males 2 females 5 females 1 male

Analyses of the less condensed metaphases revealed the presence of two types of B chromosomes. The type I B chromosomes were completely heterochromatic, and therefore, it was not possible to clearly define its morphology. In type II B chromosomes, the heterochromatic block was restricted to the centromeric region, which allowed recognition of their metacentric morphology (Fig. 2). The type I B chromosomes were found in both females and males and were present in all three populations, whereas the type II B chromosomes were found exclusively in the males from the Porto Alegre and Viçosa populations (Table 1).

**Figure 3.**
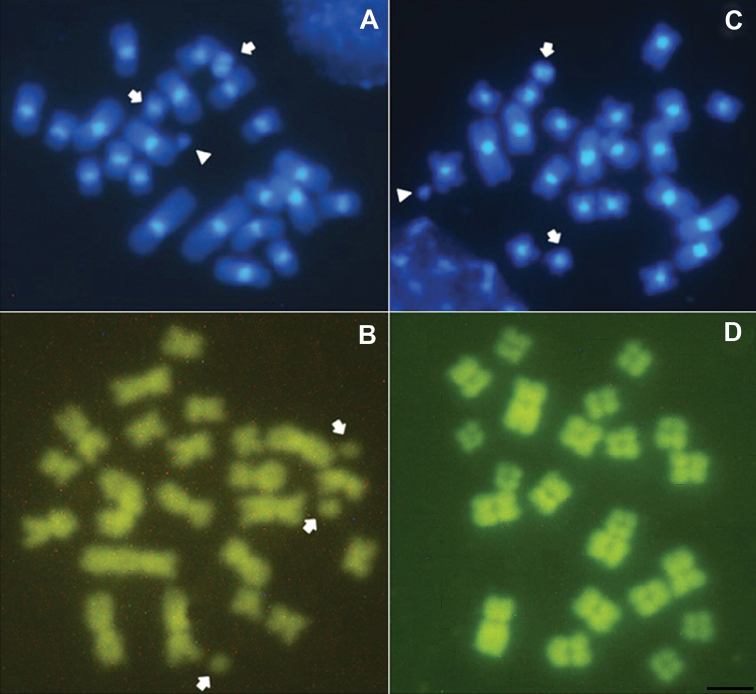
Metaphases of *Sitophilus zeamais* males (**A** and **C**) and females (**B** and **D**) stained with DAPI and CMA_3_. The arrows indicate the B chromosome, and the arrowhead indicates the Y chromosome. Bar = 10 μm.

All autosomes and the X chromosome presented small heterochromatic blocks in the centromeric region after C-banding in the four analyzed populations (Fig. 1). However, the Y chromosome of *Sitophilus zeamais* had no positive C-bands, i.e., it was entirely euchromatic. The four populations also showed DAPI^+^ bands in the centromeric region of all autosomes and the X chromosome, which coincided with the heterochromatic regions revealed with C-banding. The B chromosomes were partially or completely stained by this fluorocrome (Fig. 3A and B). No positive staining for CMA_3_ was identified in the examined populations (Fig. 3C and D).

## Discussion

The karyotypes described for the four populations of *Sitophilus zeamais* (2n = 22 chromosomes, with metacentric morphology) corroborated data for more than 42% of the 600 species of Curculionidae analyzed cytogenetically and most likely represented the ancestral karyotype of Curculionidae (Smith and Virkki 1978, Lachowska et al. 1998, 2006, 2008, Holecová et al. 2002, 2013, Rozek et al. 2009). Thus, the numerical difference found in the comparison with Barion et al. (1988), who described the karyotype of *Sitophilus zeamais* as having 2n = 20 chromosomes, as well as the differences related to the different species of *Sitophilus*, are due to intrinsic characteristics of the different techniques used.

The testes-squashing technique, commonly used in studies of Curculionidae (Rozek et al. 2004), did not allow a clear definition of chromosome morphology or an exact count of chromosomal pairs due to superposition. However, the quality of metaphases obtained in the present study with the cerebral ganglia dissociation shows clearly that this technique was effective to obtain metaphase cells with an adequate degree of condensation. Thus, this technique facilitated the determination of chromosome morphology, as well as the pairing of homologous chromosomes, and helped to overcome the difficulties encountered for the chromosome characterization of these insects. Therefore, it is also expected that with this technique it may be possible to detect chromosome differences related to size, morphology (e.g., metacentric vs. submetacentric) and the presence of secondary constrictions that facilitate distinction of closely related species.

Three of the four populations of *Sitophilus zeamais* analyzed exhibited variations in chromosome numbers due to the presence of 0–4 B chromosomes. Only in the population that originated from northern Brazil (Cruzeiro do Sul) were these chromosomes not detected. Therefore, these chromosomes apparently appeared in different populations during the expansion in the country, by different mechanisms, as they were also found in the samples analyzed by Moraes et al. (2003).

The presence of supernumerary chromosomes in Curculionidae species, however, is rare. Among more than 600 species karyotyped, only seven showed the presence of these chromosomes, *Gelus californicus* (LeConte, 1876) (Ennis 1972), *Sitophilus zeamais* (Smith and Brower 1974, Moraes et al. 2003), *Anthonomus grandis* (Boheman, 1843) (Nort et al. 1981), *Astychus* sp., *Phytoscaphus inductus* (Boheman, 1843) (Dey 1989), *Barypeithes pellucidus* Boheman 1834 (Holecová et al. 2005) and *Otiorhynchus atroapterus* (De Geer, 1775) (Holecová et al. 2013). Similar to the present study, the majority of these studies reported variations in the number of B chromosomes detected in cells, which reinforced their nonMendelian inheritance.

One difference, however, was that the B chromosomes identified in the species listed above were tiny and were similar in size to that of the Y chromosome (dot-like), whereas those identified in *Sitophilus zeamais*, though also small compared with other chromosomes in the karyotype, were clearly larger than the Y chromosome. This difference in size and the partially heterochromatic B chromosomes of *Sitophilus zeamais* evidenciated that these type II B chromosomes had a metacentric morphology, which also helped to differentiate them from the other B chromosomes previously identified in Curculionidae. Additionally, we found that *Sitophilus zeamais* females had only type I B chromosomes, whereas the males had both types. Previously, euchromatic B chromosomes were observed in only two other species of Curculionidae, *Barypeithes pellucidus* (Holecová et al. 2005) and *Otiorhynchus atroapterus* (Holecová et al. 2013). The B chromosomes were often heterochromatic in other species. Therefore, in general, the B chromosomes of *Sitophilus zeamais* possessed the same characteristics of B chromosomes of most organisms and were heterochromatic, smaller than the chromosomes of the A complement, and with a nonMendelian distribution.

Analyses of the gonadal cells of *Sitophilus zeamais* showed the “parachute” association between the X chromosome (which was relatively large) and the Y chromosome (which was the smallest chromosome of the karyotype, with a dot-like appearance). Consequently the sex determining mechanism was of the Xy_p_ type. In contrast, previous analyses defined the sex determination mechanism of *Sitophilus zeamais* as neoXY (Smith and Brower 1974). However, the results of the present study, particularly the small size of the Y chromosome, which did not show any evidence of fusion or translocation between sex chromosomes and autosomes, were in accordance with the system proposed here, which was also considered the ancestral one for this group (Smith and Virkki 1978, Lachowska et al. 1998, 2006, 2008, Lachowska and Holecová 2000, Rozek et al. 2009, Goll 2012, Holecová et al. 2013).

The C-banding patterns observed, as well as the absence of positive bands in the Y chromosome, corroborated literature data for most Curculionidae, as well as for several other insect species (Juan and Petitpierre 1989, Imai 1991, Rozek 1998, Almeida et al. 2000, Proença et al. 2002, Holecová et al. 2002, 2008, 2013, Rozek et al. 2004, Zacaro et al. 2004, Lachowska et al. 2004, 2005, 2006, 2008, 2009, Schneider et al. 2007, Lachowska 2008, Kajtoch et al. 2009). However, Curculionidae species with a large heterochromatic block on the genomes have been found (Holecová et al. 2002, Lachowska et al. 2004, 2009, Rozek et al. 2004).

Another variable aspect of the Curculionidae, when considering the heterochromatin, is the banding pattern of the Y chromosome. In many species, including *Sitophilus zeamais*, this chromosome is euchromatic; in others, such as *Centricnemus leucogrammus* Germar, 1824, *Acalles fallax*, *Acalles petryszaki* Dieckmann, 1982, *Otiorhynchus atroapterus* and *Otiorhynchus bisulcatus* (Fabricius, 1781), the presence of a completely heterochromatic Y chromosome was observed (Lachowska et al. 2006, 2009, Holecová et al. 2013). In contrast, the Y chromosome was described as with some heterochromatic regions (Lachowska et al. 2004, 2005) in *Acalles echinatus*, *Barypheites chevrolati* (Boheman, 1843), *Barypheites formaneki* (Fremuth, 1971) and *Barypheites mollicomus* (Ahrens, 1812). Thus, future studies that involve a larger number of species could demonstrate that the amount and type and location of heterochromatin regions in the chromosomes (pericentromeric, subtelomeric or intercalary) could be useful to characterize species, as well as to establish the evolutionary relationships between them (Holecová et al. 2002, 2013, Lachowska et al. 2004, 2005).

The sequential C-banding and DAPI staining performed in our study indicated that the centromeric regions of most chromosomes of *Sitophilus zeamais* were AT-rich. This result was enhanced because no CMA_3_ positive bands were identified in any of the analyzed populations. Moreover, the DAPI positive bands were often found in the same regions that were stained by C-bands in Curculionidae, which confirmed the high AT content in the heterochromatin of these insects (Lachowska 2008, Lachowska et al. 2008, Holecová et al. 2013). Lachowska (2008), Lachowska et al. (2008) and Holecová et al. (2013), for example, observed DAPI^+^ bands (pericentromeric or centromeric) on the karyotypes of six *Otiorhynchus* species, one of *Cirrohynchus* Frivaldszky, 1892, one of *Dodicastichus* (Gyllenhal, 1834), five of *Barypeithes* and two of *Strophosoma* Billberg, 1820. However, only in *Barypeithes interpositus* (Roubal, 1920), *Barypheites formaneki* (Lachowska 2008) and in *Otiorhynchus morio* (Lachowska et al. 2008) was possible to verify a weak CMA_3_^+^ band in a single autosomal pair, possibly coincident with the Nucleolus Organizer Region (NOR). According to these authors, the absence of positive signs in the other analyzed species could represent a small number of rDNA genes in the genomes. Thus, Otiorhynchus s. str. bisulcatus seemed to be an exception when comparing the fluorescent banding patterns of Curculionidae because this species showed, at the same time, positive marks for DAPI and CMA_3_ in several chromosomes. According to Holecová et al. (2013), this pattern indicated that the heterochromatin of this species consisted of repeats rich in AT and GC base pairs.

In conclusion, our cytogenetic analyses showed that the methodologies employed were effective for the characterization of the *Sitophilus zeamais* karyotype and could be further used for comparing karyotypes of other species of this genus. The karyotype of *Sitophilus zeamais* possesses features common to most species of Curculionidae, and B chromosomes are found in different populations of this species.
